# Molecular epidemiology of cattle tuberculosis in Mexico through whole-genome sequencing and spoligotyping

**DOI:** 10.1371/journal.pone.0201981

**Published:** 2018-08-23

**Authors:** Claudia Angélica Perea Razo, Elba Rodríguez Hernández, Sergio Iván Román Ponce, Feliciano Milián Suazo, Suelee Robbe-Austerman, Tod Stuber, Germinal Jorge Cantó Alarcón

**Affiliations:** 1 Doctorado en Ciencias Biológicas, Universidad Autónoma de Querétaro, Querétaro, Qro., México; 2 Centro Nacional de Investigación Disciplinaria en Fisiología y Mejoramiento Animal, INIFAP, Colón, Qro., México; 3 Facultad de Ciencias Naturales, Universidad Autónoma de Querétaro, Querétaro, Qro., México; 4 National Veterinary Services Laboratories, United States Department of Agriculture, University Blvd, Ames, Iowa, United States of America; St Petersburg Pasteur Institute, RUSSIAN FEDERATION

## Abstract

*Mycobacterium bovis* infection in cattle persists in Mexico, posing a threat to human health. Control of bovine tuberculosis, through the National Program Against Bovine Tuberculosis, has led to the decrease of disease prevalence in most of the country, except for high dairy production regions. Genotyping of *M*. *bovis* has been performed mainly by spoligotyping and variable number tandem repeats (VNTR), but higher resolution power can be useful for a finer definition of the spread of the disease. Whole genome sequencing and spoligotyping was performed for a set of 322 *M*. *bovis* isolates from different sources in Mexico: Baja California, Coahuila, Estado de Mexico, Guanajuato, Hidalgo, Jalisco, Queretaro and Veracruz, from dairy and beef cattle, as well as humans. Twelve main genetic clades were obtained through WGS and genetic diversity analysis. A clear differentiation of the Baja California isolates was seen as they clustered together exclusively. However, isolates from the central states showed no specific clustering whatsoever. Although WGS proves to have higher resolving power than spoligotyping, and since there was concordance between WGS and spoligotyping results, we consider that the latter is still an efficient and practical method for monitoring bovine tuberculosis in developing countries, where resources for higher technology are scarce.

## Introduction

*Mycobaterium bovis* is known to infect a wide variety of mammals, including the badger [1], white-tailed deer [2], brush-tailed possum [3], dogs, cats, goats, buffaloes, and pigs [4], which can act as spillover hosts to more economically important species such as dairy and beef cattle, causing bovine tuberculosis (bTB). The importance of this pathogen escalates due to its ability to infect and cause serious disease in humans [5]. In developed countries, such as Australia, Canada, the USA and New Zealand have either eradicated the disease or very nearly eradicated it, with wild fauna reservoirs preventing total eradication [6]. In Mexico, however, bTB is endemic, and although 85.8% of the national territory shows a prevalence under 0.5% [7], high milk production regions have prevalences as high as 16% [8].

To this date, studies performed on bovine tuberculosis in Mexico consist mainly on the characterization of strains through spoligotyping and VNTR [8–12], and host-pathogen interactions for immunological studies [13,14]. Furthermore, the disease in cattle is monitored through the National Program for the Control and Eradication of Bovine Tuberculosis [15], which is based on test-and-slaughter, slaughterhouse surveillance and movement restrictions for infected herds. In addition, efforts towards the control and eradication of bTB in Mexico are encouraged to improve international trade of livestock and minimize the risk to public health.

Great breakthroughs have been made with the introduction of whole genome sequencing strategies for the study of outbreaks of *M*. *tuberculosis* in humans [16–18], through which it has been possible to detect transmission chains. If this could be achieved in cattle, principal sources of infection could be identified and control efforts can be proposed in more specific directions. A few studies have already used whole genome sequencing to gain insights into the local spread and persistence of the disease in some countries [19–21]. However, in developing countries, where resources are scarce, whole genome sequencing is difficult to implement. Spoligotyping is easy to perform, cheap and requires a low amount of DNA, which is why to this date it has shown to be a useful tool for large-scale epidemiological studies, with such success that some authors consider it to be the “gold standard” [22,23]. Therefore, the aim of this study is to perform spoligotype and WGS analysis on a sub-population of *M*. *bovis* isolates from different sources in Mexico in order to compare the usefulness of each technique in this particular setting.

## Materials and methods

The study was performed mainly in Queretaro, Mexico (20.588056, -100.388056), at the Autonomous University of Queretaro. Bacterial isolates were collected in Aguascalientes (22.021667, -102.356389), Baja California (29.95, -115.116667), Coahuila (27.302222, -102.044722), Estado de Mexico (19.354167, -99.630833), Guanajuato (21.033333, -101.241667), Hidalgo (20.478333, -98.863611), Jalisco (20.566667, -103.676389), Queretaro and Veracruz (19.190278, -96.153333). Whole genome sequencing was performed at the National Veterinary Services Laboratories, in Ames, Iowa, USA (42.034534, -93.620369).

### Bacterial isolates

A total of 322 *M*. *bovis* isolates collected from cattle (n = 320) and humans (n = 2) between 1997 and 2015 in different parts of Mexico: Aguascalientes (AGS = 28), Baja California (BCA = 25), Coahuila (COA = 10), Estado de Mexico (EDOMX = 29), Guanajuato (GTO = 8), Hidalgo (HGO = 12), Jalisco (JAL = 33), Queretaro (QRO = 176), and Veracruz (VER = 1) were included in the study. This collection of isolates had previously been obtained from: bTB suspicious lesions collected from bovine carcasses at slaughterhouses and sputum samples from TB suspicious patients. After isolation from culture in Stonebrink medium [24], colonies were stored at -80° C. The protocol of this project was approved by the Bioethics Committee of the School of Natural Sciences of the Autonomous University of Queretaro, with registry number 83FCN2016.

### DNA extraction

DNA extraction was performed directly from *M*. *bovis* colonies. Briefly, colonies were thawed at room temperature for 1 hour. Then, about 100 μg of cell culture were homogenized for DNA extraction by the phenol-chloroform method (CTAB-chloroform method) [25]. Next, 50 μl of lysozyme (10 mg/ml) (Sigma Aldrich, St. Louis, Missouri, USA) was added and the sample homogenized and incubated at 37° C for 1 hour. Then 100 μl of 10% SDS was added followed by a 10 μl of a proteinase K (10 mg/ml) (Invitrogen, Carlsbad, California, USA) solution. The mixture was incubated in a warm bath at 65° C for 30 minutes. After incubation, 100 μl of 5 M NaCl was added and the mixture homogenized. This was followed by the addition of 40 μl of CTAB solution (Sigma Aldrich, St. Louis, Missouri, USA) at 10% and vortexed. The new mixture was incubated again at 65° C for 30 minutes. Following this, 400 μl of a phenol-chloroform-isoamyl alcohol reagent (volume ratio 24:24:1) was added and the mixture vortexed for 10 seconds and centrifuged at 13000 rpm for 10 minutes. From this mixture, 500 μl of aqueous phase was transferred to a 1.5 ml Eppendorf tube, where 600 μl of absolute isopropilic alcohol were added. The DNA pellet was then precipitated at -20° C for 2 hours. The product was centrifuged at 13000 rpm for 15 minutes. The supernatant was removed, and the pellet washed with 500 μl of ethanol (70%) and vortexed for 10 seconds, followed by two centrifugations at 13000 rpm. The pellet was collected and dried at room temperature for 30 minutes. The DNA pellet was then stored and frozen in 50 μl of ultra pure nuclease-free water. Final DNA concentration was 20 ng/μl, quantified with a Nanodrop^™^ 2000 (Thermofisher Scientific, Waltham, Massachussetts, USA).

### Whole genome sequencing

To obtain the whole genome sequences of the 322 *M*. *bovis* isolates, 20 ng of total DNA were used to perform sequencing on a MiSeq instrument (Illumina, San Diego, CA) using 2x250 paired-end chemistry and the Nextera XT library preparation kit (Illumina, San Diego, CA), according to manufacturer’s instructions. The bioinformatics pipeline used for this study was created by the National Veterinary Services Laboratories belonging to the United States Department of Agriculture (see https://github.com/USDA-VS; Iowa, USA), and has been used in numerous studies [18,20,26]. Quality of reads was analyzed by the software FASTQC [27]. Reads were aligned to the reference genome AF2122/97, NCBI accession number NC_0002945, using BWA and Samtools [28,29]. A depth of coverage of 100X was targeted. BAM files were processed based on Genome Analysis Toolkit (GATK)’s best practices workflow. SNPs were called using GATK’s HaplotypeCaller outputting them to variant call files (VCF) [30,31] Results were filtered using a minimum QUAL score of 150 and AC = 2. VCF files of closely related samples were grouped and SNPs were outputted to three formats: an aligned FASTA file; a formatted Excel worksheet; and a maximum likelihood phylogenetic tree created with RaxML [32] optimized for visualization using FigTree v1.4.3 (http://tree.bio.ed.ac.uk/software/figtree/). The tree was built using a GTR-CAT model with input taken as an alignment file containing only informative and validated SNPs. SNPs were also visually validated using Integrative Genomics Viewer [33,34]. Visualization of reads aligned to the reference genome AF2122/97 was performed, checking SNP’s location and coverage (S1 Fig).

### Spoligotyping and phylogenetic analysis

Spoligotypes were obtained *in silico* through the whole-genome sequence analysis performed through the NVSL in-house pipeline, as it determines the absence or presence of the spacer units in the mycobacterial genome. In order to determine evolutionary relationships among spoligotypes, a spoligoforest was built using SpolTools [35–37]. The spoligoforest provides a visualization of the probable relationships among spoligotypes in a given sample. The method makes use of a model that considers mutation by irreversible deletions of spacers and assigns probabilities to the lengths of these deletions. Solid edges are relationships between spoligotypes that are found to be unique in the data set, and the principle is that a spoligotype is inferred to arise from only one specific parent spoligotype; edges that are broken lines are chosen among multiple edges that have a probability measure greater than or equal to 0.5, and dotted lines have a probability measure less than 0.5. The size of each node is an increasing function of the number of isolates (i.e., the cluster size); edges between nodes reflect evolutionary relationships between spoligotypes with arrowheads pointing to descendants. Cladograms to establish phylogenetic relationships were made using MIRU-VNTR Plus [38,39].

## Results

Whole genome sequences of 322 *M*. *bovis* isolates from nine different states of Mexico were obtained. From these, 320 (99%) were from cattle: 217 (68%) dairy and 13 (4%) beef. No information about breed could be confirmed for 90 (28%) isolates. Two isolates (1%) were obtained from humans. The SRA sequences were deposited under NCBI Bioproject PRJNA449507. The supplemental file S1 Table lists the relevant metadata for the isolates included in this study.

### Spoligotyping

**Fig 1:**
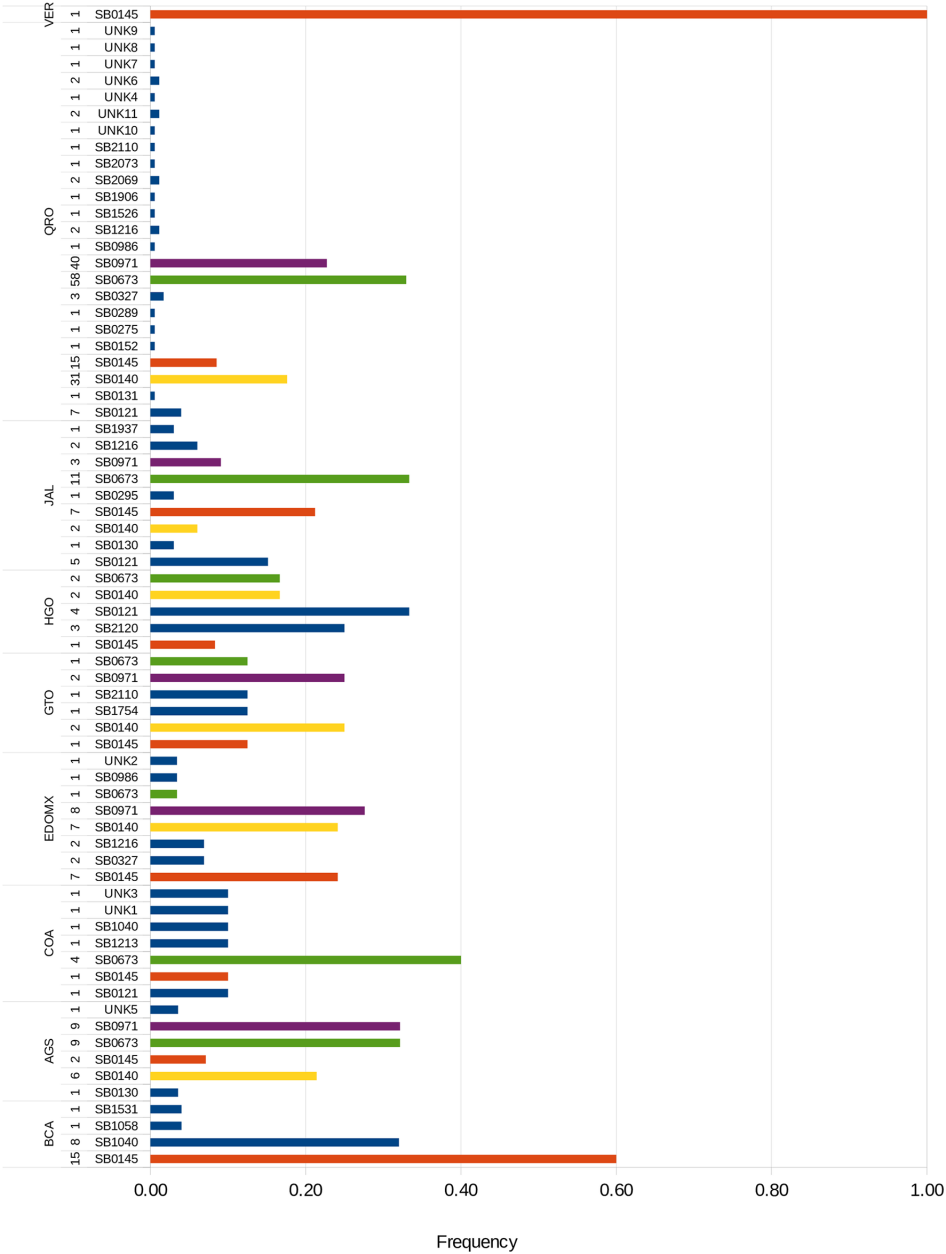
*M*. *bovis* spoligotypes. Spoligotype frequency and distribution found among the 322 *M*. *bovis* isolates. Number of isolates are presented to the left of each spoligotype. Overall most frequent spoligotypes are highlighted, green: SB0673, purple: SB0971, yellow: SB0140, and orange: SB0145. More information regarding each isolate can be found in S1 Table.

From 322 isolates, 28 recognized spoligotype patterns were identified, 11 were not found in the *Mycobacterium bovis* Spoligotype Database [40], therefore defined as “unknown” (UNK). The most frequent spoligotypes were SB0673 (85), SB0971 (60), SB0140 (50), and SB0145 (50), which included about 75% of all isolates. The distribution and frequency of each spoligotype by state is shown in Fig 1. Most spoligotypes are present in almost every state included in the study. SB0145 had the widest distribution of all, found in all the states. Unkown spoligotypes were found in COA, AGS, EDOMX, and QRO. There were two spoligotypes found exclusively in Baja California, SB1058 and SB1531. QRO had the largest genetic diversity, accounting for 24 of the 39 (61%) spoligotypes found. BCA had the lowest genetic diversity with only two spoligotypes. Spoligotypes were shared between dairy and beef cattle, as well as humans. Human isolates matched spoligotypes SB0673 and SB0971.

**Fig 2:**
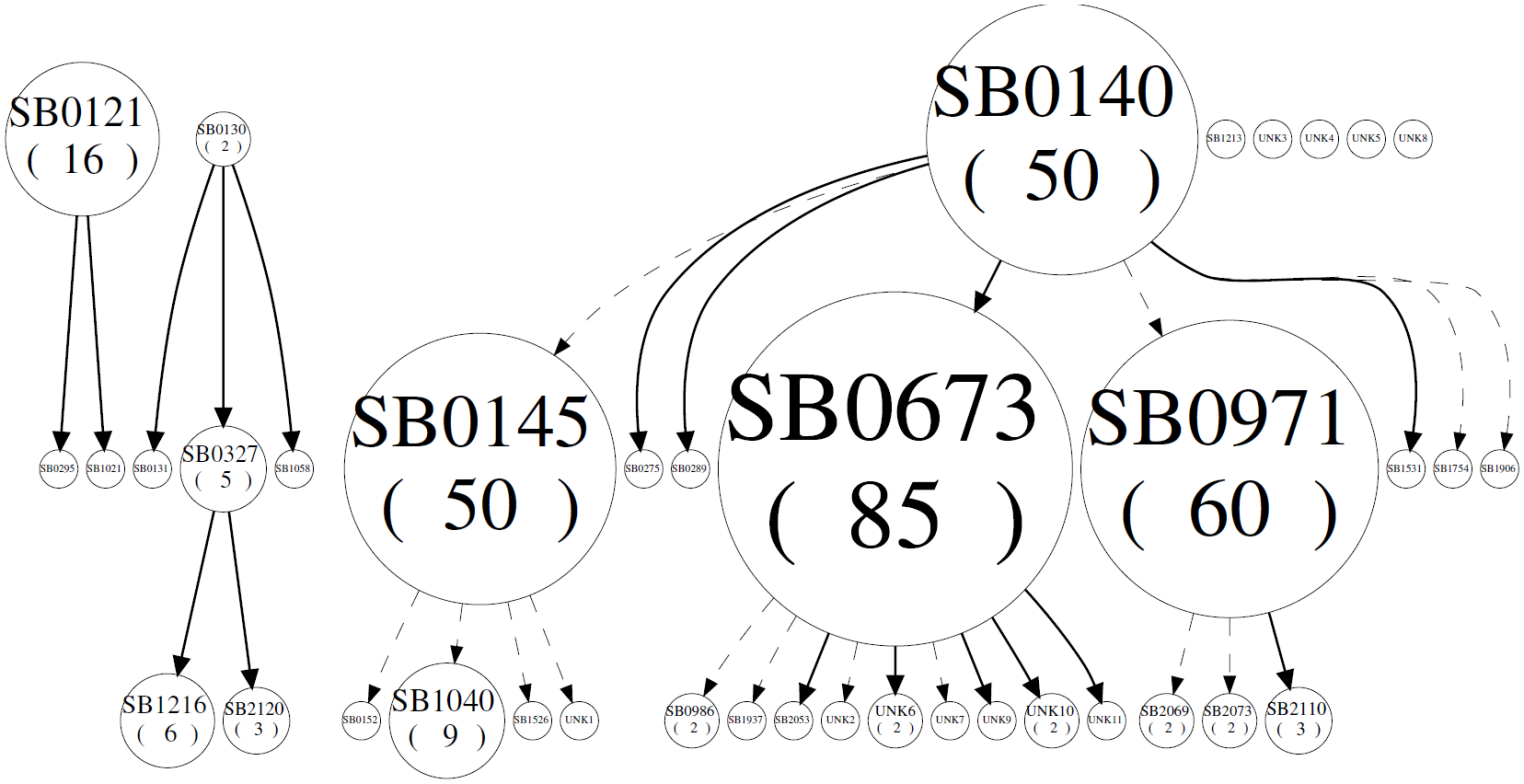
Spoligoforest of *M*. *bovis* isolates from Mexico. Evolutionary analysis by spoligoforest of the *M*. *bovis* spoligotypes obtained in this study (SpolTools).

To establish relationships between the strains, a spoligoforest was generated using the hierarchal method with a ranked layout where spoligotypes that are inferred to be derived from another spoligotype are placed below the inferred parent. In Fig 2, spoligotype SB0140 is the ancestral genotype from which most of the other spoligotypes originated, including the most frequent spoligotypes SB0673, SB0971 and SB0145. At the same time, each of these gave rise to the other less frequent extant spoligotypes. However, spoligotypes SB0130 and SB0121 form two other separate genetic groups which show no relationship with the rest. Five spoligotypes, SB1213, UNK3, UNK4, UNK5 and UNK8, had no relationship to the rest. Moreover, spoligotype SB0673, the most frequent one, is parent of nine strains, one more than the ancestral SB0140, which only gave rise to eight daughter strains.

**Fig 3:**
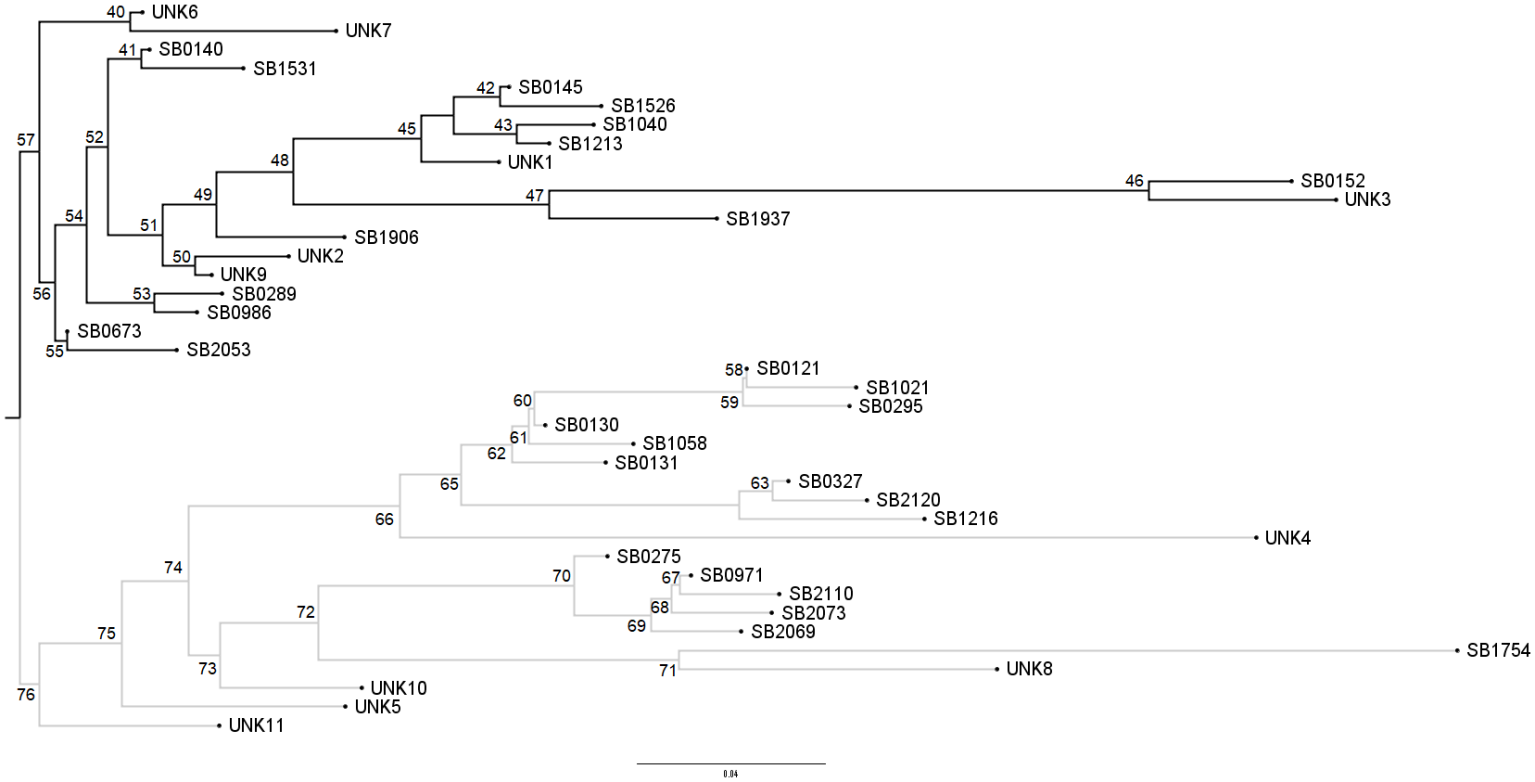
Cladogram of *M*. *bovis* spoligotypes from Mexico. Phylogenetic relationships of *M*. *bovis* spoligotypes with Neighbor-joining method using MIRU-VNTRplus.

To further resolve the genetic relationships of these strains, a neighbor-joining phylogenetic tree was generated through the MIRU-VNTRplus web application for spoligotype data (Fig 3). There are two major clades, one formed by SB0140 and its daughter strains SB0673 and SB0145, and another formed by SB0121, SB0130 and SB0971, whose relationship to SB0140 was weak through the spoligoforest analysis, represented by the dashed line. Relationships for the unknown spoligotypes were resolved, UNK3 was placed within the SB0140 clade and SB1213, UNK4, UNK5 and UNK8 were placed within the SB0121 clade.

#### SNP based genotyping and phylogenetic analysis

**Fig 4:**
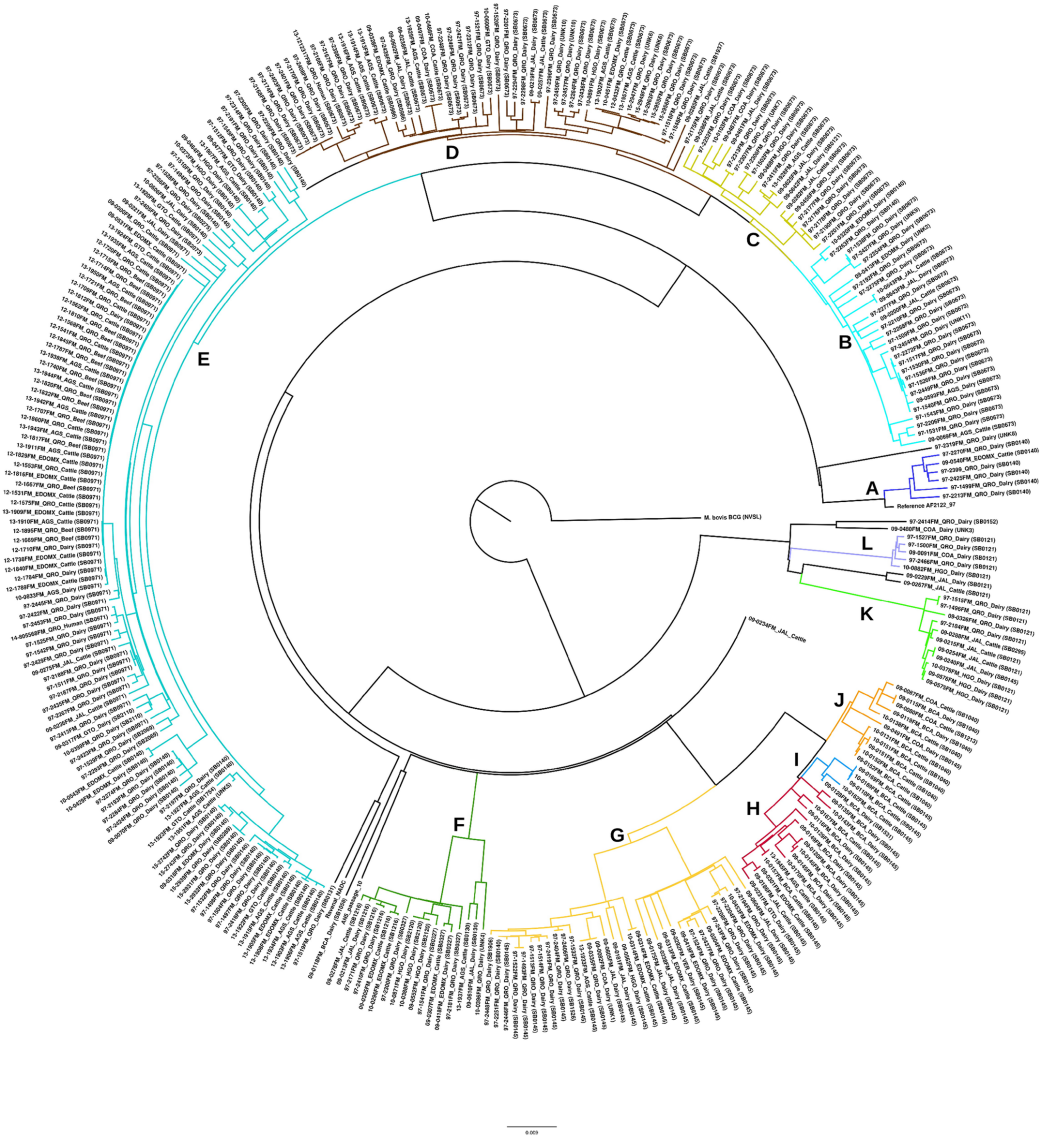
Phylogeny of *M*. *bovis* isolates. Radial representation of molecular phylogenetic analysis by Maximum Likelihood (RAxML) method based on whole-genome SNP sequences of 322 isolates of *M*. *bovis* from Mexico. Groups are represented by colors: A (blue), B (cyan), C (light green), D (brown), E (light blue), F (green), G (yellow), H (red), I (bright blue), J (orange), K (lime), L (lila).

Based on SNP data, *M*. *bovis* isolates were divided into 12 main genetic groups (Table 1 and Fig 4). The groups with the largest number of isolates were Groups E (107) and D (51). Groups D and E included isolates from dairy and beef cattle, as well as the human isolates. Group G was the most widely distributed, found in 8 of the 9 states included in the study. Group I was the least distributed, found only in BCA. Again, QRO showed the largest genetic diversity, with 9 of the 12 major SNP groups (75%). VER, on the other hand, showed the lowest genetic diversity with only major SNP Group G. Frequency and distribution of the major SNP Groups are in Table 2.

**Table 1:** SNP loci, number of isolates and spoligotypes found in each SNP Group (A-L) obtained through whole-genome SNP based analysis. (SNP sites refers to the highest number of SNPs found in each group, i.e. Group A isolates had up to 175 SNPs with respect to the reference genome AF2122/97).

Major SNP Group	SNP sites	No. of isolates	Spoligotype
**A**	175	6	SB0140
**B**	572	28	SB0140, SB0673, UNK9, UNK11, UNK2
**C**	378	21	SB0121, SB0673, UNK7
**D**	750	51	SB0673, SB0986, SB1937, UNK6, UNK10
**E**	1418	107	SB0140, SB0275, SB0289, SB0971, SB1754, SB2069, SB2073, SB2110
**F**	451	16	SB0130, SB0327, SB1216, SB2120, UNK4
**G**	986	32	SB0145, UNK1
**H**	123	11	SB0145
**I**	54	4	SB0145, SB1531
**J**	190	11	SB0145, SB1040, SB1213
**K**	95	11	SB0121, SB0145, SB0295
**L**	63	5	SB0121

Clustering of isolates did not result from host species, breed or year of isolation. However, there is evidence of clustering by geographic location with respect to the isolates from BCA, which form three SNP Groups, H, I and J. At leas one “unknown” strain is found within each group.

**Table 2:** Distribution of each major SNP Group by state. Number of isolates corresponding to a specific group within a state is indicated in parenthesis (). It is possible that the total number of isolates per state does not equal the sum of the isolates per group because not all isolates fell within a group, some were outliers in the phylogeny (i.e. 09-0234FM_JAL_Cattle).

Origin	AGS	BCA	COA	EDOMX	GTO	HGO	JAL	QRO	VER
Total number of isolates per state*	28	25	10	29	8	12	33	176	1
**SNP Group**	B (1) C (1) D (6) E (16) F (1) G (1) H (1)	H (11) I (5) J (8)	C (2) D (2) G (1) J (3) L (1)	A (1) B (2) D (2) E (4) F (4) G (6) H (1)	D (1) E (6) H (1)	C (1) D (1) E (2) F (3) G (1) K (3) L (1)	B (3) C (5) D (4) E (4) F (3) G (5) H (1) K (6)	A (5) B (21) C (12) D (35) E (69) F (6) G (18) K (4) L (3)	G (1)

## Discussion

Bovine tuberculosis in Mexico remains an endemic disease [9], which leads to the restriction of international trade of cattle and cattle products, and most importantly, poses a threat to public health. To be able to monitor the disease, genetic profiling of the pathogen is necessary. Spoligotyping and WGS are efficient for the molecular characterization of *M*. *bovis*, though the latter provides more information relevant to the pathogen’s biology [41], while spoligotyping is useful for diagnosis and strain characterization [42]. Unfortunately, WGS is still a high-cost and complex technology that many developing countries can’t yet fully incorporate it into routine disease surveillance activities. In this study we aimed at comparing both methods in order to evaluate the capacity of spoligotyping to stand against WGS to and continue to be a valid method for bovine tuberculosis surveillance.

In Mexico, typing of *M*. *bovis* has been done by RFLP, RAPD-PCR, spoligotyping, and MIRU-VNTR [8,10,11,43,44]. Spoligotyping is cheap, easy to perform, it requires minimal amount of DNA and there is an international database where spoligotypes can be reported, stored and compared [Smith et al., 2012]. However, against WGS, this method has poor discriminatory power and it is prone to homoplasy, where unrelated strains evolve independently. In our results, spoligotyping grouped the strains into 28 genetic groups, of which the most frequent were SB0673, SB0971, SB0140, SB0145 and SB0121. These spoligotypes have been found previously at high frequency in Mexico [9,45–49]. In order to establish the relationships among the spoligotypes and classify groups of related strains, a spoligoforest (Fig 2) and a cladogram (Fig 3) were built. Overall, there are two main clades of *M*. *bovis*, SB0140 and SB0121, and their daughter strains. These correspond to groups A-J and K-L, respectively. A recent study in Eritrea [50] clustered *M*. *bovis* isolates into two groups that also corresponded to spoligotype pattern. In Uruguay [51], similar results were obtained, they found concordance between clustering by spoligopyte and WGS, obtaining three main *M*. *bovis* clades in the territory of Uruguay.

Through whole genome sequencing, however, it is possible to follow the evolution of a strain according to the SNPs it acquires over time. This way, strains that are a few SNP differences apart could be considered directly related. From our study have been able to see how strains of spoligotypes SB0971 and SB0673 have diverged into numerous subgroups. Spoligoforest analysis also places SB0971 and SB0673 as parent strains of a few spoligotypes (Fig 2), though resolution is poor. Due to the higher resolving power of WGS, spoligotypes overlap among two or more different groups in the SNP phylogeny (Fig 4), such as SB0673, which can be seen in Groups B, C and D. In Table 1, the same is seen for other spoligotypes: SB0121 in Groups C, K and L; SB0145 in Groups G through K; SB0121 in Groups K and L; and SB0140, because it is an ancestral genotype from which the majority derived, in Groups A, B and E. Finally, it is important to highlight that cattle and humans share spoligotypes SB0673 and SB0971, and SNP-types D and E, respectively. Other authors have found similar results with respect to human and bovine isolates [48,52], suggesting clear transmission between hosts.

It is clear that the discriminatory power is much higher with WGS than with spoligotyping; however, the cost is also considerably higher and hard to accomplish for large amounts of isolates in large-scale studies in developing countries. WGS is worth the cost for studies of evolution or for tracing back sources of infection in the presence of outbreaks. Perhaps in large-scale epidemiological studies where the objective is only to monitor disease prevalence, such as in medium to high-burden countries like Mexico, where there is great genetic diversity, spoligotyping is efficient and accurate. Although spoligotyping is not considered cutting-edge technology any more, it is still used worldwide for the study of bovine tuberculosis, mainly in developing countries where resources for WGS technology are scarce [50,53–60].

According to the number of spoligotypes and WGS typing groups, it seems that spoligotyping is more discriminatory than WGS, since there are 28 spoligotypes and only 12 major SNP groups. In WGS, every isolate that does not match perfectly should be identified as different, so there may be only 28 spoligotypes, but since each isolate has a different SNP profile, then there are 322 genotypes. In this study, we counted a group as one that contained all isolates that were within sharing a common ancestor by 10 to 20 SNPs. For example, major SNP group H includes 11 isolates, grouped into spoligotype SB0145, but it can be divided into 6 subgroups, where three subgroups have two isolates each, and the rest are single-isolate subgroups. In some cases, WGS is so sensitive that it provides very detailed information that can be complex to interpret. For this reason, spoligotyping may be adequate for large-scale epidemiological studies.

## Conclusion

Spoligotyping and WGS complement each other. WGS is an excellent tool for detecting sources of infection and transmission chains during outbreak investigations, but it remains a costly and complex procedure for developing countries to incorporate into routine disease surveillance activities. Spoligotyping, though not of recent date, continues to be a valid technique for the molecular characterization of *M*. *bovis* strains worldwide. WGS requires important resources, financial, computational and technical. Spoligotyping is easy and cheap to perform, so it is still a legitimate method for large scale epidemiological studies. In Mexico, more studies should be performed that include isolates from all of the states in order to get a more precise picture of the dynamics of bovine tuberculosis in the country.

## Supporting information

S1 Fig Visual validation of SNPs with Integrative Genomics Viewer (IGV). Reads are aligned to the reference genome AF2122/97, coverage is represented as a gray bar chart in the upper track of each strain and SNPs are shown as colored bars.
(TIF)

S1 Table Sequence metrics and metadata. Metrics and metadata for the 322 *M*. *bovis* whole genome sequences used in this study.
(XLS)
