# Supplementary figures and images for: Molecular epidemiology of cattle tuberculosis in Mexico through whole-genome sequencing and spoligotyping
Source: PLoS One. 2018 Aug 23;13(8):e0201981. doi: 10.1371/journal.pone.0201981 (PMC6107157; doi:10.1371/journal.pone.0201981)

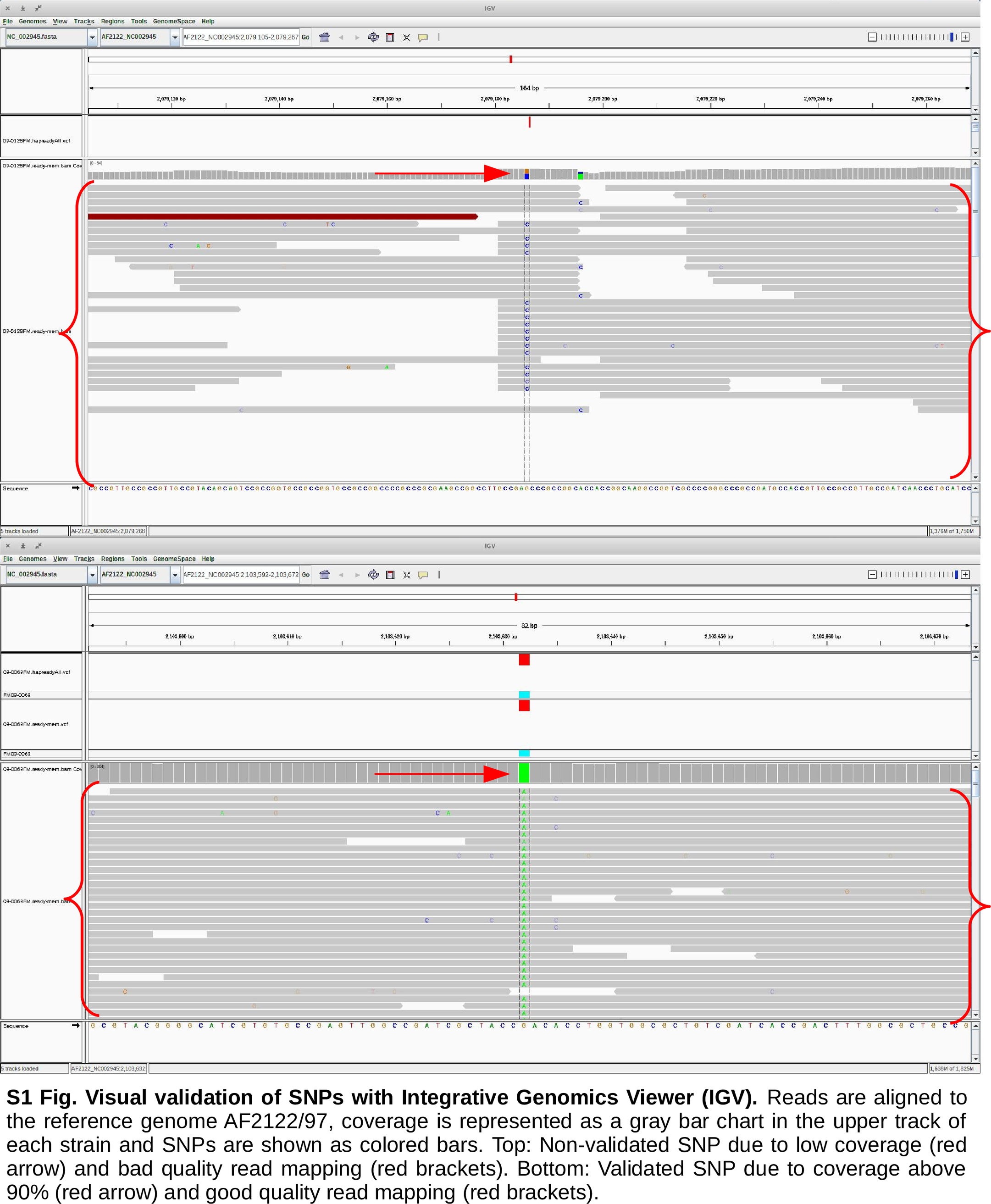

Supplement: S1 Fig — Reads are aligned to the reference genome AF2122/97, coverage is represented as a gray bar chart in the upper track of each strain and SNPs are shown as colored bars. (TIF) [file pone.0201981.s001.tif]
